# Nurturing People and Planet: Breastfeeding in Climate Mitigation and Adaptation

**DOI:** 10.3389/fsufs.2026.1919608

**Published:** 2026-09-15

**Authors:** Justin E. Silpe, Karla Damian-Medina, Sarah M. Reyes, Bonnie L. Bassler

**Affiliations:** 1 PumpKin Baby Inc, Princeton, New Jersey, USA; 2 Rev Bioscience, LLC, Boise, Idaho, USA; 3 Princeton University, Princeton, New Jersey, USA; 4 Howard Hughes Medical Institute, Chevy Chase, Maryland, USA

**Keywords:** Breastfeeding, Climate change, Environmental sustainability, Carbon footprint, Food security, Maternal health, Policy integration, Microplastics

## Abstract

Infant feeding practices have environmental consequences that are largely absent from climate policy discussions. Breastfeeding relies on maternal metabolism and local support systems, whereas commercial milk formula depends on dairy production, industrial processing, packaging, and extensive supply chains. Comparative analyses report higher greenhouse-gas and water burdens for commercial milk formula than for breastfeeding. Globally, commercial milk formula use among infants under 6 months of age adds roughly 5.9–7.5 billion kg of carbon dioxide equivalent annually. Pumping and bottle-feeding add material, electricity, and cleaning-related burdens relative to feeding at the breast, but available evidence generally indicates lower impacts than commercial milk formula, although estimates depend strongly on assumptions about maternal diet and pumping practices. Climate change and water insecurity can also threaten breastfeeding through heat stress, food insecurity, and disruptions during disasters and emergencies. Moreover, uncontrolled formula donations during upheavals, while well-intended, impede continuation of breastfeeding. Collectively, these pressures can generate a reinforcing feedback loop: climate shocks undermine breastfeeding, increasing reliance on resource-intensive substitutes that further strain water and energy systems. Recognizing breastfeeding as both a mitigation and resilience issue highlights co-benefits for maternal and child health and climate action and points to practical policy levers, including paid maternity leave, workplace lactation protections, responsible marketing of commercial milk formula, and continuity of lactation support during emergencies that are needed to align optimal infant feeding with climate adaptation planning.

## 1 Introduction

The climate crisis demands examination of all human activities contributing to environmental degradation, including the often-overlooked domain of infant feeding. Historically, breastfeeding has been promoted primarily for its maternal and child health benefits: reducing infant mortality, preventing infections, supporting optimal child development, and reducing maternal risks of breast and ovarian cancer (Victora et al., 2016). Only recently has the environmental dimension of infant feeding emerged as a consideration for planetary health (Rollins et al., 2016). This Perspective calls for breastfeeding to be incorporated into environmental assessments and climate policy and for breastfeeding support to be strengthened as part of climate-resilience planning.

The urgency of this reframing stems from converging global trends. Despite WHO recommendations for exclusive breastfeeding for the first six months of life, fewer than half of infants meet this benchmark. The 2025 Global Breastfeeding Scorecard reports 47% exclusive breastfeeding among infants under 6 months (World Health Organization and UNICEF, 2025). Global commercial milk formula sales reached approximately US$55.6 billion in 2019 (Baker et al., 2021). Here, commercial milk formula refers to manufactured breastmilk substitutes, including infant, follow-on, and toddler formulas. Commercial marketing can shape infant-feeding practices (Zhu et al., 2023), while the production and use of these products carry environmental costs through greenhouse-gas emissions, water consumption, and waste generation, impacts that remain largely absent from climate policy discussions (Smith, 2019).

Equally concerning is the threat climate change poses to breastfeeding. Rising temperatures, extreme weather events, and disasters and emergencies create physiological and social barriers to successful lactation (Traylor et al., 2025). For environmental scientists and policymakers unfamiliar with lactation physiology, it is essential to understand that breastfeeding is a biological food system in which maternal metabolism supports infant nutrition (Butte and King, 2005). Breastfeeding avoids most of the industrial production, packaging, transport, and preparation inputs required by commercial milk formula, although successful breastfeeding still depends on maternal health, time, and social support (Smith, 2019). These differences help explain breastfeeding’s environmental advantages while also highlighting its dependence on supportive social and health systems. Breastfeeding support therefore spans health, labor, regulatory, and emergency systems, including skilled lactation care, paid leave, workplace accommodations, implementation of the International Code of Marketing of Breast-milk Substitutes, and emergency planning (IFE Core Group, 2017; World Health Organization and UNICEF, 2017). This piece draws on evidence from life-cycle assessment, climate science, and maternal-child health considerations to examine breastfeeding as both a climate mitigation and a climate adaptation strategy. Here, we synthesize quantitative estimates and policy-relevant examples to inform climate and public health planning.

## 2 The Environmental Footprint of Infant Feeding

### 2.1 Quantifying the Impact

Published estimates of the environmental impact of infant feeding vary because studies use different functional units, geographies, and system boundaries. Some analyses include maternal dietary intake and household practices, whereas others focus more narrowly on commercial milk formula production, household preparation, or feeding accessories. These methodological differences should be kept in mind when comparing numerical estimates across studies (Amonkar et al., 2019; Andresen et al., 2022; Cos-Busquets et al., 2025; Karlsson et al., 2019; Smith et al., 2024b).

Nonetheless, several comparative analyses report higher greenhouse-gas burdens for commercial milk formula than for breastfeeding or mixed feeding, although the magnitudes of the differences can change when maternal diet and pumping practices are modeled explicitly (Amonkar et al., 2019; Andresen et al., 2022; Cos-Busquets et al., 2025; Karlsson et al., 2019; Smith et al., 2024b). Karlsson et al. (2019) estimated that replacing breastfeeding with commercial milk formula for the first 6 months of life was associated with 95–153 kg CO_2_e per infant, depending on the country setting. In a Norwegian life-cycle assessment, 4 months of exclusive commercial milk formula feeding produced 35–72% higher impacts than exclusive breastfeeding across five environmental categories (Andresen et al., 2022). At a population level, the Green Feeding Climate Action Tool estimates that commercial milk formula use among infants under 6 months of age contributes roughly 5.9–7.5 billion kg CO_2_e annually worldwide (Green Feeding Tool, n.d.; Smith et al., 2024b).

Notably, a substantial share of the commercial milk formula burden comes from follow-on and “growing-up” products marketed for older infants and toddlers, categories that major health agencies consider unnecessary for most children. Although intensive marketing campaigns suggest otherwise, WHO guidance notes that follow-up formulas are not required for healthy infants and young children when appropriate complementary foods are introduced alongside continued breastfeeding (World Health Organization Regional Office for Europe, 2019). In a life-cycle analysis of commercial milk formula sold in six Asia Pacific countries, projected 2017 emissions from milk formula sold in China alone exceeded 4 million tons of CO_2_e, and follow-up and toddler formula products accounted for the largest share of sales-driven emissions growth (Dadhich et al., 2021).

Greenhouse-gas emissions from the commercial milk formula industry arise primarily from dairy production, where enteric methane from cattle digestion contributes substantially to global greenhouse-gas emissions. Many commonly used infant formulas rely heavily on bovine-milk ingredients and agricultural inputs for feed production that require fertilizers that emit nitrous oxide (Pope et al., 2021; Smith, 2019). Formula manufacturing also contributes carbon dioxide emissions through energy-intensive processes such as pasteurization, spray-drying, and packaging. Global supply chains carrying ingredients and finished products add transportation emissions, and home preparation requires energy for boiling water and sterilizing equipment.

Differences in water consumption also distinguish breastfeeding from formula feeding. Producing commercial milk formula powder requires several thousand liters of water when dairy production, feed crops, and manufacturing are incorporated into calculations, although exact estimates vary according to whether blue, green, and gray water are considered and how household use is modeled (Hoekstra, 2008; Pope et al., 2021; Smith et al., 2024b). Reconstitution at home requires additional water for mixing, bottle washing, and, in many settings, sterilization. Using the Green Feeding Climate Action Tool, commercial milk formula consumed by infants under 6 months of age requires about 2,562.5 billion liters of water annually (Green Feeding Tool, n.d.; Smith et al., 2024b).

### 2.2 The Environmental Impact of Breastmilk Pumping

Direct breastfeeding has the lowest environmental burden among human-milk feeding methods. Many mothers provide breastmilk through pumping, storage, and bottle-feeding, all of which add material, electricity, and cleaning demands. The GREEN MOTHER study reported a median climate-change impact of 0.01 kg CO_2_e per day per infant for exclusive breastfeeding when feeding accessories were assessed, however, the authors note that their reported infant-feeding totals did not include maternal diet impacts (Cos-Busquets et al., 2025). Amonkar et al. (2019) modeled a U.S. pumping scenario that did include maternal caloric intake as well as pumping equipment, storage, and cleaning. Under maximal assumptions, the study estimated higher daily greenhouse-gas emissions for the modeled pumped-breastmilk scenario than for commercial formula feeding. Notably, 66% of the emissions in that model derived from the mother’s additional caloric intake, the validity of which other experts have questioned (Cos-Busquets et al., 2025; Smith et al., 2024b). For context, exclusive breastfeeding typically increases maternal dietary energy needs by approximately 450 kcal/day (Butte and King, 2005). Taken together, these studies suggest that pumping increases the environmental burden relative to direct feeding at the breast, but the magnitude depends strongly on assumptions about maternal diet, storage, electricity use, and household practice (Amonkar et al., 2019; Becker and Ryan-Fogarty, 2016; Cos-Busquets et al., 2025).

The water footprint of exclusive pumping is likewise context dependent. Unlike feeding at the breast, pumping and bottle-feeding require routine washing of pump components and bottles and, depending on context, periodic sterilization. These activities require additional household water, although actual use varies substantially by cleaning method, pumping frequency, appliance efficiency, and access to running water (Becker and Ryan-Fogarty, 2016). Commercial formula feeding also depends on safe household water for mixing and hygiene and carries a much larger upstream water footprint from formula production (Green Feeding Tool, n.d.; Hoekstra, 2008; Smith et al., 2024b). Beyond climate issues, pumped milk retains the immunological and nutritional benefits of human milk, making it an important alternative when direct breastfeeding is not feasible.

**Figure 1.**
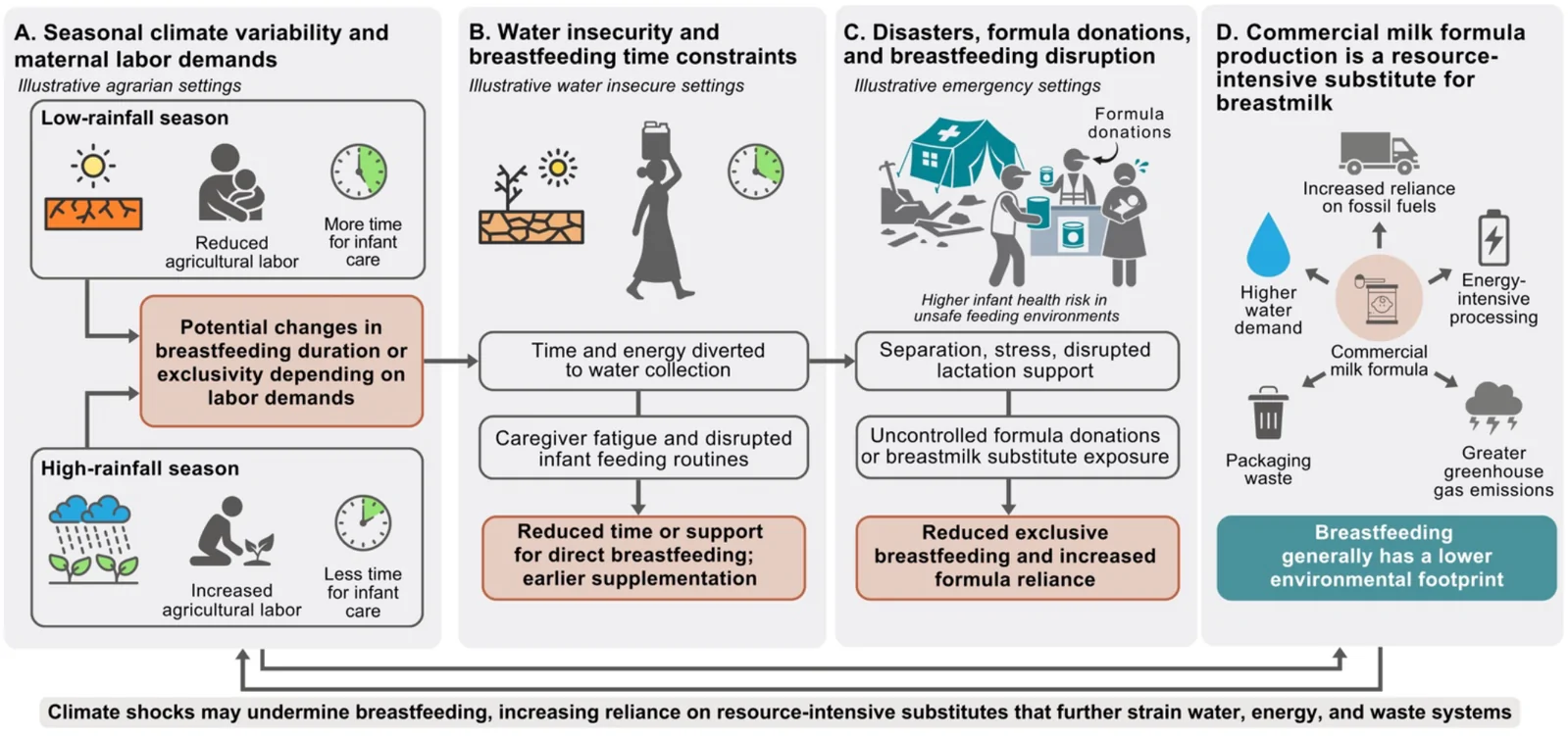
Infant feeding, environmental footprint, and climate-related vulnerability pathways. (A) Breastfeeding and commercial milk formula feeding differ in resource requirements and environmental burdens. Breastfeeding relies on maternal metabolism and local support systems. Commercial milk formula production depends on the dairy industry, industrial processing, packaging, transport, and household preparation. Published estimates report higher greenhouse-gas, water, and waste burdens for commercial milk formula compared to breastfeeding, although values vary by system boundary and assumptions. (B) Water insecurity can divert caregiver time and energy, disrupt infant feeding routines, and increase reliance on commercial milk formula in settings where safe commercial milk formula preparation may be difficult. (C) Disasters can separate mothers from infants, disrupt lactation support, and increase commercial milk formula dependence, particularly when donations are uncontrolled. (D) Climate-related stressors can reduce breastfeeding exclusivity, duration, or both, increasing reliance on commercial milk formula and adding pressure to water, energy, and waste systems. Figure 2 expands the pathways by which climate-related stressors can disrupt breastfeeding and increase commercial milk formula use.

Waste generation further distinguishes human milk feeding from formula feeding (Figure 1). Commercial formula feeding generates packaging and feeding-equipment waste, including metal tins, paperboard, plastics, bottles, teats, and single-use accessories (Pope et al., 2021; Smith, 2019). Direct breastfeeding generates little or no packaging waste at the point of feeding, converting maternal resources directly into infant nutrition.

### 2.3 Hidden Environmental Costs of Commercial Formula Feeding

Beyond direct production impacts, commercial formula feeding can generate additional, often unconsidered emissions. Compared with breastfed infants, formula-fed infants experience higher rates of some respiratory and gastrointestinal infections, which can increase healthcare use and associated energy and material consumption (Bartick and Reinhold, 2010). Marketing and retail distribution also require resources such as advertising production, transport, and retail infrastructure that are typically excluded from environmental assessments. While these indirect contributions are difficult to quantify, they reinforce the point that infant feeding decisions impinge on larger commercial and healthcare systems that possess their own environmental footprints. These unquantified pathways should therefore be treated as research gaps rather than included in current comparative estimates.

## 3 Climate Change as a Threat to Breastfeeding

### 3.1 Food and Water Security

**Figure 2.**
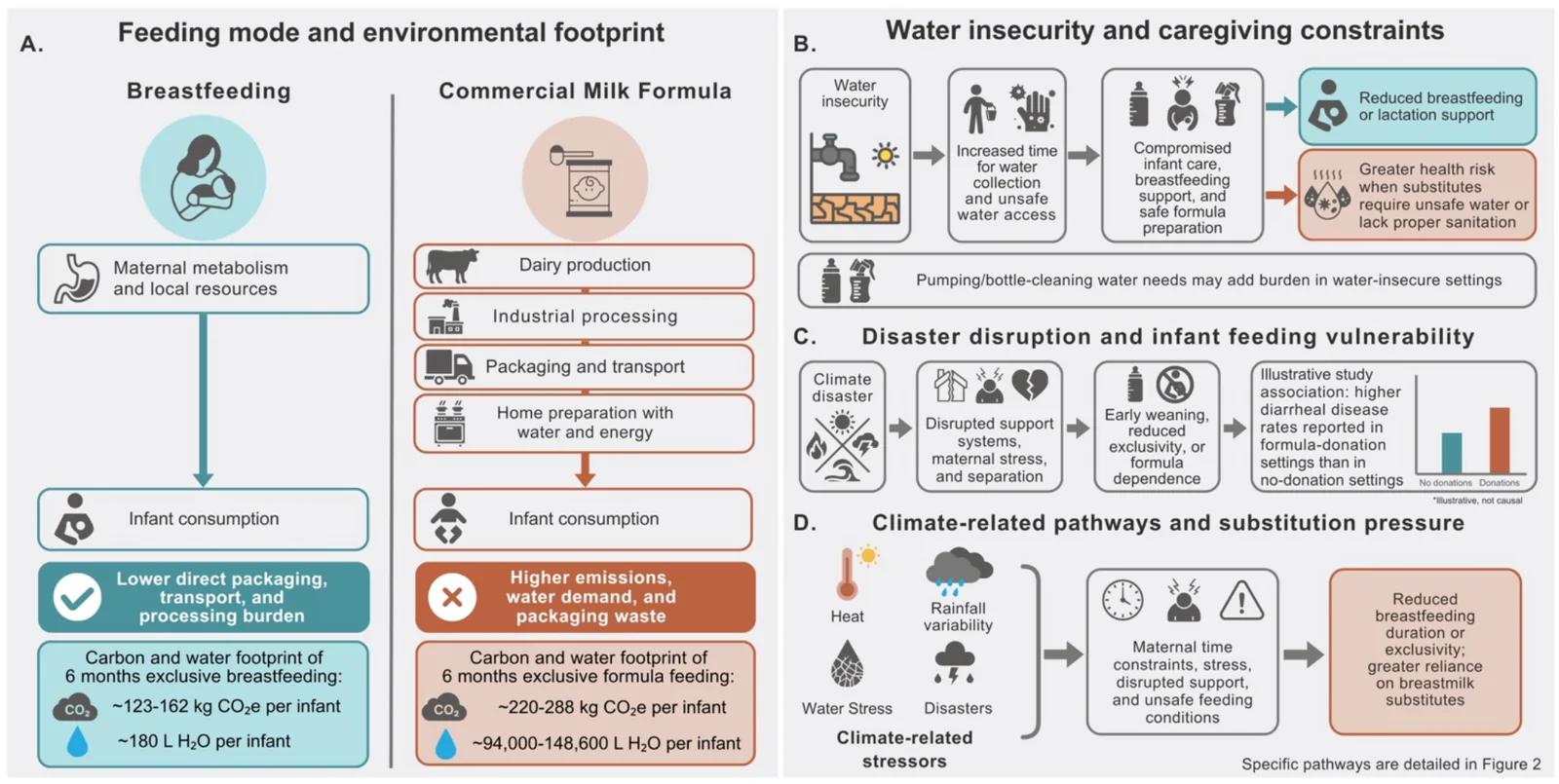
Climate-related issues that, without breastfeeding support interventions, may disrupt breastfeeding and increase reliance on commercial milk formula. (A) Seasonal climate variability can alter maternal labor demands and infant caregiving time, affecting breastfeeding opportunity and duration. (B) Water insecurity can redirect maternal time and energy toward water collection and household management, disrupting infant feeding routines and increasing the likelihood of earlier supplementation with commercial milk formula. (C) Disasters and emergency responses can separate mothers from infants, increase stress, interrupt lactation support, and promote commercial formula use, most notably when commercial milk formula donations are uncontrolled. (D) Compared with feeding at the breast, increased reliance on commercial milk formula amplifies demand for dairy production, safe water, energy-intensive processing, transport, packaging, and waste management. Together, these issues illustrate how climate shocks can undermine breastfeeding while increasing dependence on commercial milk formula along with its higher environmental footprint.

Climate change threatens infant feeding through its impacts on food and water systems. Drought-afflicted regions show direct correlations between hunger seasons and maternal nutritional stress, with mothers perceiving reduced milk quality during periods of inadequate caloric intake. While some mothers resort to early weaning, most continue breastfeeding with supplementation despite these challenges (Grace et al., 2017; Webb-Girard et al., 2012). While moderate malnutrition typically does not affect breastmilk production quantity, severe deficits can reduce breastmilk production and alter breastmilk composition, driving a cascade of vulnerabilities where climate-induced food insecurity affects the youngest members of society most severely. The relationship between climate variability and breastfeeding practices has been empirically demonstrated in rural Ethiopia, where greater rainfall during the kiremt season was associated with reduced exclusive breastfeeding duration (a 20-percentage-point decrease in the likelihood of six-month exclusive breastfeeding when comparing 25 cm versus 5 cm of average monthly rainfall), as peak rainfall intensifies agricultural labor demands, forcing mothers into the fields and disrupting feeding schedules (Figure 2A) (Randell et al., 2021).

Water scarcity presents a paradox for breastfeeding mothers in resource-limited regions. Across 24 sub-Saharan African countries, an estimated 13.54 million adult women and 3.36 million children were responsible for water collection in households where collection exceeded 30 minutes. This time burden directly competes with infant care, potentially delaying breastfeeding or disrupting feeding schedules (Figure 2B) (Graham et al., 2016). Research from water-insecure communities across 19 sites in 16 LMICs found that water scarcity negatively influenced infant breastfeeding practices and infant health (Schuster et al., 2020). Cultural misconceptions about hydration needs during lactation persist, with some mothers believing excessive water intake improves milk production, despite evidence showing mothers need only drink to thirst (Ndikom et al., 2014). This misunderstanding can lead to unnecessary supplementation with commercial formula when water is scarce, counterproductively further straining water demands while undermining the protective benefits of exclusive breastfeeding. UNICEF’s 2023 Climate Changed Child supplement estimates that 739 million children worldwide were living in areas of high or very high water scarcity in 2022 (UNICEF, 2023).

### 3.2 Environmental Pollutants and Breastmilk Quality

Beyond climate-related stressors, environmental pollutants represent an additional layer of complexity for breastfeeding in a changing world. Air pollution, water contaminants, persistent organic pollutants, endocrine-disrupting chemicals, and heavy metals can accumulate in maternal tissues and transfer to breastmilk (Serreau et al., 2024). These exposures may alter maternal milk production and/or composition, potentially affecting lactation success and raising concerns about infant exposure through breastfeeding.

Current evidence generally supports continued breastfeeding even in polluted environments. Commercial formula feeding does not eliminate infant exposure to environmental contaminants, and commercial formula powders, packaging, and the water used for preparation can introduce their own contaminant exposures depending on the setting. A critical review of the epidemiological literature found no consistent or clinically relevant health consequences to infants exposed to background levels of environmental chemicals in breastmilk, while noting that research on chemicals in commercial formula and infant health outcomes remains limited (LaKind et al., 2018). Breastmilk contains immunological components, antioxidants, and bioactive factors that may help mitigate some adverse effects of environmental pollutants such as air pollution, although findings vary across exposures and outcomes (Zielinska and Hamułka, 2019). This finding underscores the importance of addressing pollution at its source through environmental policy rather than discouraging breastfeeding, which could compound environmental and health harms.

Concerns about environmental contaminants sometimes lead caregivers to view commercial formula as a “cleaner” alternative to breastmilk, but microplastics illustrate the opposite trade-off. Microplastic particles have been detected in powdered infant formulas, with one analysis estimating that exclusively formula-fed infants could ingest on the order of tens of particles per day from the powder itself (Kadac-Czapska et al., 2024). The larger source may stem from feeding equipment. Polypropylene bottles, which dominate the infant feeding bottle market, can shed microplastics, especially when exposed to high temperatures during sterilization, with reported releases in the millions of particles per liter under standardized conditions (Li et al., 2020). Compared with feeding directly at the breast, any form of bottle-feeding (whether formula or expressed milk) can therefore increase infant exposure to microplastics, particularly when bottles are heated. Microplastics have also been detected in human milk, but breastmilk feeding avoids many upstream plastic contacts associated with industrial manufacturing, packaging, and high-temperature reconstitution (Liu et al., 2023).

### 3.3 Disasters and Emergencies

Disasters and emergencies, including floods, hurricanes, wildfires, and earthquakes, can threaten breastfeeding through multiple mechanisms. Separation of mothers from infants during evacuations disrupts feeding routines and can trigger early weaning. Stress from displacement and trauma can suppress the milk ejection reflex, while loss of privacy and support systems in emergency shelters creates practical barriers to breastfeeding. Uncontrolled commercial formula donations can further undermine breastfeeding continuation when it is most critical for infant survival (IFE Core Group, 2017). Recent emergencies have exemplified these challenges, including earthquakes in Indonesia and Türkiye, where unsolicited formula donations and code violations were documented (Guan et al., 2024; Hipgrave et al., 2012). For instance, after the 2006 Yogyakarta earthquake, 80% of households with infants received unsolicited formula donations, and one-week diarrhea incidence among recipient infants reached 25.4% versus 11.5% among non-recipients, a relative risk of 2.12 (Figure 2C) (Hipgrave et al., 2012). Similarly, during the 2006 Botswana diarrheal disease outbreak, non-breastfed infants experienced significantly higher hospitalization and mortality rates than did breastfed infants, further demonstrating how commercial formula dependence amplifies vulnerabilities during emergencies (Creek et al., 2010). High-income settings are not exempt: in the United States, Hurricane Helene in western North Carolina in 2024 disrupted power, water, pumping, milk storage, and lactation support, and the Support and Advocacy in Feeding Emergencies (SAFE) program was established in response, training relief organizations to manage thousands of pallets of donated infant formula by prioritizing ready-to-feed products and discarding expired stock (Anderson et al., 2025). Such scenarios highlight the need for breastfeeding-protective emergency protocols that prioritize lactation support and carefully managed infant feeding assistance over indiscriminate commercial formula distribution, as outlined in the Operational Guidance on Infant and Young Child Feeding in Emergencies (IFE Core Group, 2017). Recent reviews document breastfeeding as optimal during disasters but highlight persistent preparedness gaps (Gribble et al., 2019).

## 4 The Bidirectional Relationship between Breastfeeding and Climate Change: Implications for Health and Environment

A complex bidirectional relationship exists between breastfeeding and climate change, with critical implications for both health and environmental resilience. Climate change threatens breastfeeding by undermining food security, maternal health, and water access, ironically weakening the very practice that could reduce the environmental footprint of infant feeding. This connection generates a reinforcing feedback loop: environmental degradation reduces breastfeeding, leading to increased commercial formula use, which further strains natural resources and drives increased emissions (Meadows, 2008).

Within this bidirectional relationship, cascading effects amplify population-level vulnerabilities that compound over time. In regions like Pakistan, where breastfeeding is associated with markedly lower infant mortality, protecting breastfeeding remains especially important when climate shocks, food insecurity, or displacement increase infant vulnerability (Amir-ud-Din et al., 2022). The resulting shift to commercial formula feeding also increases dependence on safe water, fuel, and sanitation for preparation and cleaning, resources that may be constrained during climate shocks.

These cascading effects are particularly evident in water-stressed settings. In sub-Saharan Africa, the time burden of water collection can compete with infant care and feeding schedules, potentially disrupting breastfeeding (as noted above and in Figure 2B) (Graham et al., 2016; Schuster et al., 2020). Paradoxically, when reduced breastfeeding leads to greater reliance on commercial formula, household water needs increase further for mixing and hygiene, perpetuating a cycle that undermines both infant health and community resilience to climate change.

Conversely, high breastfeeding rates can enhance community resilience to climate shocks by reducing dependence on supply chains, safeguarding infant nutrition, and lowering water and energy use (Grubesic and Durbin, 2022). Thus, breastfeeding can contribute both to mitigation goals, by reducing reliance on resource-intensive substitutes, and to resilience, by supporting infant feeding when infrastructure is disrupted. Protecting breastfeeding may therefore create a reinforcing cycle: resilient communities are better able to support breastfeeding, and breastfeeding, in turn, can reduce dependence on fragile first-food systems (Joffe et al., 2019).

Supporting and protecting breastfeeding represents a practical, low-cost climate action with immediate benefits. In comparative estimates based on 6 months of feeding, breastfeeding can avoid emissions on the order of 95–153 kg CO_2_e per infant relative to commercial formula, although the precise value depends on methods and assumptions (Karlsson et al., 2019). This reduction can coincide with nutritional and immunological benefits for the infant, including during environmentally stressful conditions.

## 5 Future Directions and Research Needs

The emerging connection between breastfeeding and climate change mitigation requires expanded research across multiple domains to strengthen evidence and guide optimal health recommendations and policy implementation. Several critical areas demand immediate attention.

Complete life-cycle assessments including all commercial formula types, pumping equipment, and production methods are needed to generate comparable environmental coefficients for distinct feeding scenarios. Phase I of the GREEN MOTHER project has provided comprehensive environmental impact data for different infant feeding regimes (Cos-Busquets et al., 2025), however, specific comparisons between exclusive pumping and direct breastfeeding remain understudied. Quantifying healthcare-related emissions from feeding modes and modeling population-level impacts of breastfeeding rate changes on national emissions would provide needed data. Modeling should link national data on births and feeding patterns to harmonized life-cycle coefficients and compare current practices with breastfeeding-target scenarios. The Green Feeding Climate Action Tool offers a starting framework; future models should incorporate pumping, mixed feeding, local energy and water conditions, healthcare use, and uncertainty (Green Feeding Tool, n.d.; Smith et al., 2024b). Expanded assessments should also examine marketing, retail, and last-mile distribution. Standardized methodologies for measuring water consumption during pump cleaning routines need development, as current estimates rely on equipment guidelines rather than on empirical data.

Climate adaptation research must also develop and test interventions that support lactation during heat stress. As extreme heat events become more common, understanding the physiological and social thresholds at which heat exposure affects milk production, maternal hydration, and infant feeding practices will become increasingly important. A recent systematic review and meta-analysis highlights the broader risks of heat exposure for maternal and neonatal health and underscores the need for targeted protections for pregnant and postpartum people (Lakhoo et al., 2025). In that pooled analysis of 198 studies across 66 countries, each 1 °C rise in ambient temperature was associated with an odds ratio of 1.04 (95% CI 1.03 to 1.06) for preterm birth, while heatwave exposure was associated with an odds ratio of 1.26 (95% CI 1.08 to 1.47). Research should evaluate practical strategies such as access to cooling, rest breaks, hydration protocols, and workplace accommodations that help mother-infant pairs continue breastfeeding during hot weather and during heat-related disruptions.

The intersection of environmental pollutants and breastfeeding requires nuanced investigation. While current evidence generally supports continued breastfeeding in polluted settings (LaKind et al., 2018; Zielinska and Hamułka, 2019), a more precise understanding of how to minimize contaminant exposure while maintaining breastfeeding remains important. Studies should examine how different pollutants affect milk production and composition and whether particular interventions can reduce pollutant transfer to breastmilk while preserving its beneficial properties. Additionally, optimal dietary and lifestyle modifications need to be defined for lactating mothers in contaminated environments.

Scalable interventions linking climate change and breastfeeding messaging require implementation. How do mothers respond to environmental information about infant feeding choices? Do climate co-benefits to breastfeeding motivate behavior change or institutional support? Real-world pilots demonstrating feasibility and impact will be essential for scaling successful approaches. Research should also explore the effectiveness of integrating environmental messaging into existing breastfeeding promotion programs and whether framing breastfeeding in terms of climate action influences policymaker support. Developing monitoring systems that track both breastfeeding rates and associated environmental impacts could accelerate progress.

Technological innovations may also offer solutions to reduce the environmental impact of necessary breastmilk pumping. Research into more efficient cleaning methods, reusable storage systems, and pumps designed for minimal water use could help mothers who need to pump while minimizing resource consumption and climate impact. Similarly, investigating traditional breastmilk storage methods from various cultures might reveal sustainable alternatives to current plastic-intensive systems. Implementation research should test how paid leave, workplace accommodations, skilled lactation care, Code implementation, emergency preparedness, and proposed climate-finance mechanisms affect breastfeeding outcomes and the environmental impacts estimated from those outcomes (Smith et al., 2024a; World Health Organization and UNICEF, 2017).

## 6 Conclusion

The climate crisis requires attention to emissions and resource use across sectors, including infant feeding. Commercial milk formula carries substantial environmental impacts through dairy production, processing, packaging, and transport. By contrast, breastfeeding is a low-resource infant feeding system with a comparatively small footprint. Heat stress, water insecurity, and disasters and emergencies can disrupt breastfeeding by affecting maternal health, separating families, and interrupting support services. When breastfeeding declines, reliance on commercial formula can rise, increasing exposure to supply-chain disruptions and further straining water and energy systems. Protecting and supporting breastfeeding is therefore relevant to both climate change mitigation and climate change adaptation.

Moving forward in the breastfeeding and climate change arena requires breaking down artificial boundaries between health and environmental sectors. Health, labor, and social-protection systems can contribute to climate goals by protecting and supporting breastfeeding through skilled lactation care, paid leave, and workplace accommodations, which can reduce reliance on resource-intensive commercial milk formula. Instruments such as the Green Feeding Climate Action Tool illustrate that even modest increases in breastfeeding can translate into meaningful emissions and water savings at the population level (Green Feeding Tool, n.d.; Smith et al., 2024b). Emergency planners should also treat lactation support as an essential service in disasters and emergencies, recognizing that commercial formula dependence during crises can increase infection risk and may be dangerous where water and sanitation are compromised (Creek et al., 2010; Hipgrave et al., 2012; IFE Core Group, 2017).

This perspective calls on researchers, practitioners, and policymakers across disciplines to collaborate in positioning breastfeeding as an essential component of climate-resilient, sustainable development. Human and planetary health are inseparable, and infant feeding sits at that intersection. Enabling mothers to initiate and maintain breastfeeding through supportive policies and services can benefit maternal and child health while reducing dependence on resource-intensive first-food systems.

## Data Availability

No original datasets were generated or analyzed for this article.

## List of Abbreviations:

CO_2_e: Carbon Dioxide Equivalent
LMICs: Low- and Middle-Income Countries
WHO: World Health Organization
UNICEF: United Nations Children’s Fund
